# Supplementary figures and images for: Comparison of observational methods to identify and characterize post-COVID syndrome in the Netherlands using electronic health records and questionnaires
Source: PLoS One. 2025 Jan 29;20(1):e0318272. doi: 10.1371/journal.pone.0318272 (PMC11778627; doi:10.1371/journal.pone.0318272)

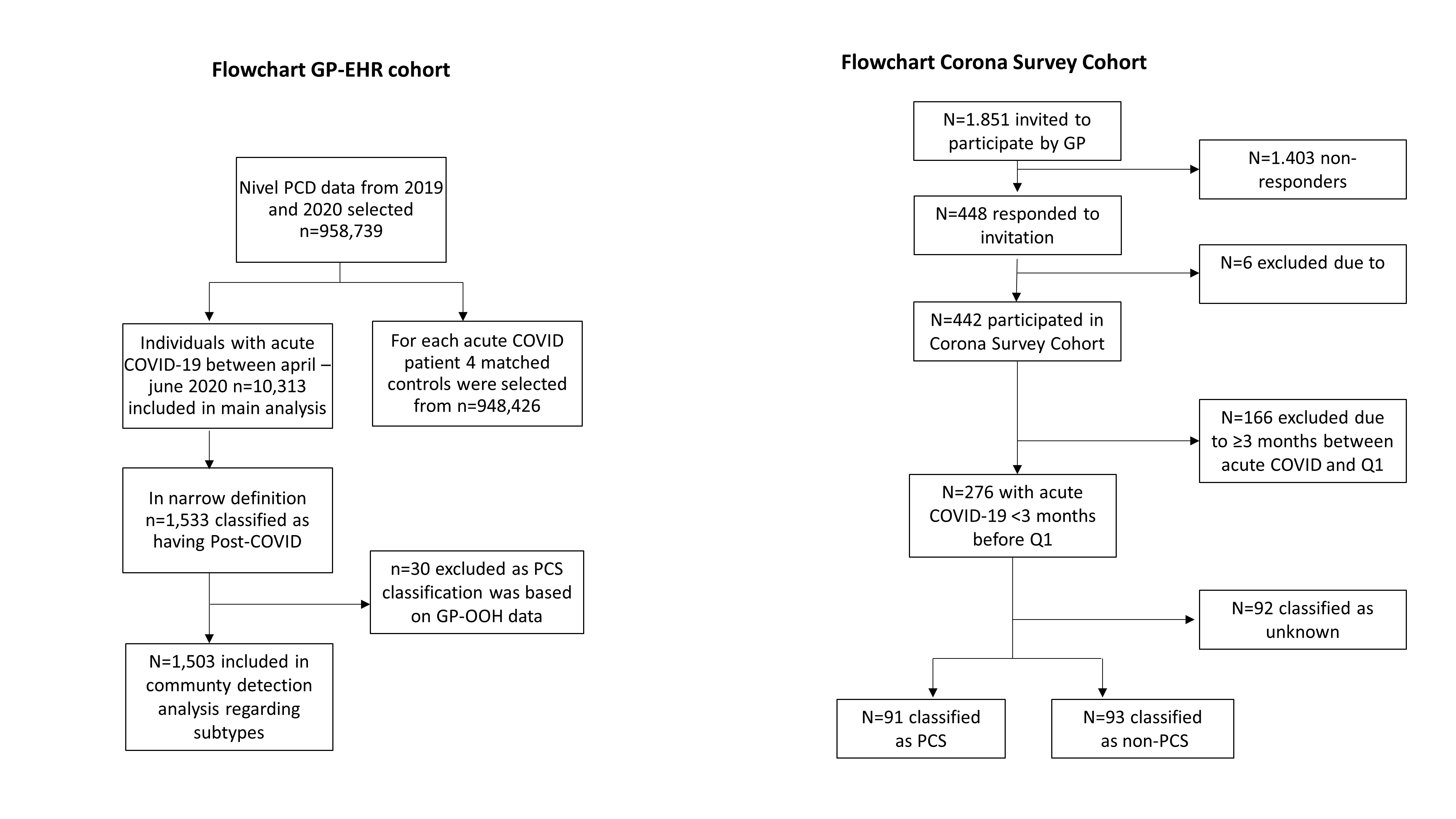

Supplement: S1 Fig — Schematic overview of the included study populations of the GP-EHR cohort and the Corona Survey Cohort. (PNG) [file pone.0318272.s001.png]
