# Supplementary material for: Comparison of observational methods to identify and characterize post-COVID syndrome in the Netherlands using electronic health records and questionnaires
Source: PLoS One. 2025 Jan 29;20(1):e0318272. doi: 10.1371/journal.pone.0318272 (PMC11778627; doi:10.1371/journal.pone.0318272)
Supplement: S1 Table — Demographic and socioeconomic characteristics of the matched reference group that was used for classification. (DOCX) [file pone.0318272.s002.docx]

| S1 Table. Characteristics of matched control group | | |
| --- | --- | --- |
|  | | Matched control group (no COVID) |
| n (% of total COVID-19 group) | | 41,244 |
| Age (mean, SD) | | 51.5 (19.5) |
|  | No. children and adolescents | 1,804 (4.4) |
|  | No. adults | 31,849 (77.2) |
|  | No. elderly | 7,591 (18.4) |
| Male, n (%) | | 16,948 (41.1) |
| Low education level, n (%) | |  |
|  | High | 8,529 (20.7) |
|  | Medium | 6,911 (16.8) |
|  | Low | 9,771 (23.7) |
|  | Unknown | 16,033 (38.9) |
| Household income, n (%) | |  |
|  | High | 7,814 (18.9) |
|  | Medium | 15,802 (38.3) |
|  | Low | 15,749 (38.2) |
|  | Unknown | 1,879 (4.6) |
| Migration background, n (%) | | 8,517 (20.7) |

Categories were classified as follows: children and adolescents (age 0 – 23 years of age), adults (24 – 70 years of age), elderly (≥ 70 years of age). Migration background was dichotomized as: both parents were born in the Netherlands (0) and at least one parent is not born in the Netherlands (1). Education level was divided into low (primary school of pre-vocational education), medium (secondary or vocational education) and high (professional higher education or university) education level. Income level was divided according to standardized household income in the Netherlands into low (0-40 percentile), medium (40-80 percentile) and high (>80 percentile).
