# Supplementary material for: Comparison of observational methods to identify and characterize post-COVID syndrome in the Netherlands using electronic health records and questionnaires
Source: PLoS One. 2025 Jan 29;20(1):e0318272. doi: 10.1371/journal.pone.0318272 (PMC11778627; doi:10.1371/journal.pone.0318272)
Supplement: S2 Table — Symptoms and diagnosis are based on ICPC coding and are classified as core or additional symptom. Symptoms and diagnosis are also classified in categories which are used in Fig 1. (DOCX) [file pone.0318272.s003.docx]

S2 Table. List of core and additional symptoms and diagnosis used in GP-EHR cohort

| **Symptom/Diagnosis** | **Type of symptom** | **Categories** |
| --- | --- | --- |
| Fever | Core | Fever |
| Tiredness/weakness | Core | Fatigue/reduced fitness |
| Nausea | Core | Dizzy/nauseous |
| Vomit | Core | Dizzy/nauseous |
| Diarrhea | Core | Abdominal complaints |
| Ringing in the ears/tinnitus | Core | Hearing complaints |
| Muscle strain | Core | Muscle pain/joint pain |
| Headache | Core | Headache |
| Other peripheral neuritis/neuropathy | Core | Neurological complaints |
| Crisis/transient stress response | Core | Psychological complaints |
| Insomnia/other sleep disorder | Core | Sleep complaints |
| Dyspnoea/tightness of the chest attributed to airways | Core | Respiratory complaints |
| Other breathing problems | Core | Respiratory complaints |
| Cough | Core | Coughing/sneezing/stuffy nose |
| Sneezing/nasal congestion/runny nose | Core | Coughing/sneezing/stuffy nose |
| Throat symptoms/complaints | Core | Throat and voice complaints |
| Acute upper respiratory tract infection | Core | COVID-19 |
| Influenza (ex. R81) | Core | Influenza (ex. R81) |
| Other respiratory infection(s) | Core | COVID-19 |
| Generalized pain | Additional | Generalized pain |
| Perspiration problems | Additional | Excessive sweating |
| Allergy/allergic reaction | Additional | Allergy/allergic reaction |
| Fear of other illness | Additional | Fear of COVID-19 |
| Other general symptoms/complaints | Additional | Other general symptoms/complaints |
| Other viral disease(s) | Additional | Other viral disease(s) |
| Other infectious disease(s) | Additional | Other infectious disease(s) |
| Abnormal test result(s). | Additional | Abnormal test result(s). |
| Other generalized/unspecified disease(s) | Additional | Other generalised/unspecified disease(s) |
| Enlarged lymph node(s) | Additional | Enlarged lymph node(s) |
| Pernicious/folic acid deficiency anemia | Additional | Pernicious/folic acid deficiency anaemia |
| Generalized abdominal pain/abdominal cramps | Additional | Abdominal complaints |
| Heartburn | Additional | Abdominal complaints |
| Other localized abdominal pain | Additional | Abdominal complaints |
| Constipation | Additional | Abdominal complaints |
| Change in stool/defecation pattern | Additional | Abdominal complaints |
| Red eye | Additional | Red eye |
| Hearing complaints (ex. H84,H85,H86) | Additional | Hearing complaints |
| Pain attributed to heart | Additional | Heart complaints |
| Palpitations/awareness of heartbeat | Additional | Psychological complaints |
| Paroxysmal tachycardia | Additional | Heart complaints |
| Chest symptoms/complaints | Additional | Heart complaints |
| Hand/finger symptoms/complaints | Additional | Extremity complaints |
| Symptoms multiple/unspecified joints | Additional | Muscle pain/joint pain |
| Tension headache | Additional | Headache |
| Restless legs | Additional | Neurological complaints |
| Tingling fingers/feet/toes | Additional | Extremity complaints |
| Vertigo/dizziness (ex. H82) | Additional | Dizzy/nauseous |
| Migraine | Additional | Headache |
| Feeling anxious/nervous/tense | Additional | Psychological complaints |
| Memory/concentration/orientation disorders | Additional | Cognitive complaints |
| Other psychological symptoms/complaints | Additional | Psychological complaints |
| Anxiety disorder/anxiety state | Additional | Psychological complaints |
| Depression | Additional | Psychological complaints |
| Personality/character disorder | Additional | Psychological complaints |
| Other symptoms/complaints nose | Additional | Coughing/sneezing/stuffy nose |
| Symptoms/complaints voice | Additional | Throat/voice complaints |
| Fear of other respiratory diseases | Additional | Fear of COVID-19 |
| Other respiratory symptoms/complaints | Additional | Respiratory complaints |
| Acute/chronic sinusitis | Additional | Coughing/sneezing/stuffy nose |
| Acute tonsillitis/ peritonsillar abscess | Additional | Acute tonsillitis/ peritonsillar abscess |
| Acute bronchitis/ bronchiolitis | Additional | Respiratory complaints |
| Pneumonia | Additional | Respiratory complaints |
| Asthma | Additional | Respiratory complaints |
| Hyperventilation | Additional | Hyperventilation |
| Hair loss/alopecia | Additional | Hair loss/alopecia |
| Fat metabolism disorder(s) | Additional | Fat metabolism disorder(s) |
| Bladder malignancy | Additional | Bladder complaints |
| Painful menstruation | Additional | Menstrual complaints |
| Irregular/frequent periods | Additional | Menstrual complaints |
| Intermenstrual bleeding | Additional | Menstrual complaints |
| Loss/death of partner | Additional | Psychological complaints |
