# Supplementary material for: Comparison of observational methods to identify and characterize post-COVID syndrome in the Netherlands using electronic health records and questionnaires
Source: PLoS One. 2025 Jan 29;20(1):e0318272. doi: 10.1371/journal.pone.0318272 (PMC11778627; doi:10.1371/journal.pone.0318272)
Supplement: S3 Table — Demographic and socioeconomic characteristics of individuals in the Corona Survey Cohort who could not be classified and were therefore excluded from the analyses. (DOCX) [file pone.0318272.s004.docx]

S3 Table. Characteristics of Unknown group in Corona Survey Cohort

|  | |  |  |
| --- | --- | --- | --- |
| **Characteristics** | | | **Unknown group** |
| n (% of total Corona Survey Cohort) | | | 92 (33) |
| Age (mean, SD) | | | 45.6 (14.7) |
| Male, n (%) | | | 42 (45.7) |
| Level of education | | |  |
|  | Low, n(%) | | 16 (17.4) |
|  | Medium, n(%) | | 28 (30.4) |
|  | High, n(%) | | 30 (32.6) |
|  | Unknown, n(%) | | 18 (18.6) |
| Migration background, n (%) | | | 4 (4.4) |
| No. of self-reported symptoms | | |  |
|  | After 3 months (n=192) | |  |
|  | After 6 months (n=160) | | 3.7 (2.9) |
| No. of individuals who are working less or stopped working | | |  |
|  | After 3 months (total n=185) | |  |
|  | After 6 months (total n=147) | |  |
