# Supplementary material for: Comparison of observational methods to identify and characterize post-COVID syndrome in the Netherlands using electronic health records and questionnaires
Source: PLoS One. 2025 Jan 29;20(1):e0318272. doi: 10.1371/journal.pone.0318272 (PMC11778627; doi:10.1371/journal.pone.0318272)
Supplement: Attachment — Submitted filename: PONE-D-24-29474_Response to Reviewers.docx [file pone.0318272.s005.docx]

Manuscript-ID: PONE-D-24-29474

Title: Comparison of observational methods to identify and characterize Post-COVID syndrome in the Netherlands using electronic health records and questionnaires

We would like to thank the reviewers for their thorough reading and constructive comments. This has helped us to further improve our manuscript. The reviewers have primarily asked for further clarifications and textual adjustments. Below we addressed the comments and questions raised by the reviewers. We hope that the manuscript is now suitable for publication in PLOS ONE.

**Point-by-point response**

**Reviewer 1**

1) **The major issue with the Corona Virus survey is that it was not possible to determine for around a third of the sample whether or not they had PCS. Given this, and what is probably a low response rate I am not convinced that estimates of frequency from this sample are particularly meaningful.**

There were indeed n=92 (33%) participants in the Corona Survey Cohort classified as ‘unknown’ due to missing data or discrepancies in their survey answers. We also described this in the manuscript (page 7, lines 22-24) to be transparent about the choices we made in this study. Survey research, like every research method, has its limitations. Despite the limitations, the frequency estimates based on the survey are valuable because in our manuscript we were able to compare them directly to estimates based on electronic health records and to discuss discrepancies and similarities and their implications. The aim of this manuscript was to provide more insights on the post-COVID syndrome but also to demonstrate the influence of using different research methods in epidemiological research in general. We have now elaborated on this more in the Introduction of the manuscript to substantiate our choices and thereby also addressing comment 3 (page 4, lines 2-8). In addition, we have also described this limitation of using survey data in the Discussion (page 15, lines 16-19).

2) **There is also lack of clarity and transparency around the sample size used in analyses. As far as I can tell the analytic sample for the main analyses using EHRs was10,313. This is stated in the results. This should probably be the figure stated in the abstract. Instead the authors state 1,000,000 in the abstract and 50,000 at the start of the discussion section. The authors should provide a flow chart indicating how the analytic sample is derived and in the abstract and discussion state the analytic sample not the size of the population in the database from which it was derived. Similarly, the authors should consider a flow chart indicating how the final analytic sample for the Corona Virus Survey was derived. I think the proportion of the sample that has been classified into the three clusters by exploratory network analysis is only 10%. If this interpretation is correct my conclusion would be that this method is not very useful.**

   As suggested by the reviewer we have created a flowchart to clarify sample sizes in the different groups and analyses which we will add as an Appendix to the manuscript (Supplementary Figure 1). We have adjusted the Abstract and the Discussion as it now states that n=10.313 individuals were used for the main analysis.

Regarding the community detection analysis, these analysis were conducted in n=1503 individuals. Even though this is a smaller subgroup we do find these analysis useful as we explored whether data driven techniques could be helpful to create subgroups based on symptoms registered in GP-EHR data. The added value of these analysis is a twofold: 1) for post-COVID it is still uncertain which subgroups or phenotypes actually exist as literature points in different directions and therefore these analysis provide new insights on this matter, 2) using this technique with EHR data is a proof-of-concept of the methodology (community detection analysis) to determine whether it could be useful in other settings or topics. Based on this we do believe that these analysis are insightful, but we agree that the results should be interpreted with caution and this is also mentioned in the Discussion section (page 13, lines 26-28).

3) **The introduction should also include more on the theoretical basis of the difference methods used to Classify PCS in the study and why they might be useful.**

We have now elaborated on this in the Introduction of the manuscript as the operationalization of different classifications and methods is crucial for research into post-COVID syndrome but also epidemiological research in general (please see manuscript page 4, lines 2-8).

4) **Could the authors check that all references are complete. Several including 22, 28 and 38 are not. There may be other references that are incorrect.**

We have now checked and adjusted the references.

5) **The data access statement is vague. The authors say data is available by request form the author, but also that is restricted by the use of special licenses. The authors need to make it much clearer what data is available, and the process required to apply for it. I would be very surprised if the EHR data is available on request, as generally there are many restrictions put in place to limit risk of disclosure. In addition, there is a substantial body of research showing when authors state “data is available on request” in practice it is often very hard if not impossible to get hold of the data.**

As this was also stated by the Editorial Office we have already adjusted the Data Access statement. Please find the adjusted statement below.

*Data requests for usage of data from Nivel Primary Care Database or the Corona Survey Cohort may be submitted to the applicable governance bodies via gegevensaanvragen@nivel.nl. In this manuscript data from Nivel Primary Care Database was linked to data available at Statistics Netherlands and we used the Microdata Platform of Statistics Netherlands as a secure research environment. The linked database is only available at the Microdataplatform of Statistics Netherlands and can be made accessible if certain conditions are fulfilled.*

6) **In the abstract the authors should state the number of ways in which PCS has been defined and given that there does not appear to be very many, list the frequency of PCS symptoms for each method.**

We have now added the number of ways in which PCS was defined and the frequency of symptoms for which the GP was consulted to the Abstract.

7) **Also in the abstract, I found the description of “exploratory network analysis” confusing. Normally, I would associate network analysis with social networks of people, not as a method of investigating clusters and that needs to be clear.**

We have adjusted this as it now states ‘community detection analysis’ in both the method and result section of the Abstract.

8) **On page 4; Could the authors clarify the source used to derive socio-demographic data. Does it come from the GP records or is it via linkage from a different source?**

The socio-demographic data originates from various sources, but are provided through Statistics Netherlands and linked to the GP-EHR on the Microdata platform from Statistics Netherlands. We have now rephrased this in the Manuscript to make it more clear (please see page 4, lines 24-26).

9) **Page 6; Line 3: Could you please provide a bit more detail on how patients were flagged by GPs as having Covid-19. I am not familiar with the Dutch system so don’t know how this would be done.**

General practitioners (GPs) are required to record a diagnostic code (ICPC code) for every consultation. During the COVID pandemic a new diagnostic code was desohmated for acute COVID: ICPC code R83.03. By recording this code, individuals who consulted their GP with acute COVID could be identified. We have now rephrased this in the manuscript to make it more clear (page 6, line 8).

10) **Page 6 to 7: There is a single paragraph spanning more than a page. It would be easier to read and understand the different definitions of PCS if this paragraph was broken up into different sections.**

This paragraph has now been divided into three sections to enhance readability.

11) **Page 7, Line 14: “Individuals were classified as having PCS when they reported at least one symptom, from a selected list of symptoms” If this is the list of symptoms in supplementary table 2 this should be explicitly stated, if it is a different list of symptoms, it should be included as an additional supplementary table. In addition, the n for the number of individuals classified as having PCS is not clear in the text.**

    We have now mentioned in the manuscript that indeed the list of symptoms is in Supplementary Table 2. The numbers classified as having PCS are listed in Table 1.
12) **The discussion section would be improved if the reasons for a very large difference between PCS and Non-PCS with respect to the proportions working less than 3 months 23% versus 0%) were discussed.**

We have now elaborated on this finding regarding work (dis)ability and PCS in the Discussion (see Manuscript page 13, line 28-29 – page 14 line 1-8) as follows:

*We found that in the Corona Survey Cohort that 23% of the individuals with PCS stopped working or worked less after three months compared to 0% in the non-PCS group. After six months 11% of individuals with PCS stopped working or worked less. It has been reported in other studies as well that work ability can be severely affected by PCS which may have large consequences at individual but also on societal level [38-40]. Tailored interventions for PCS in relation to work focusing on management of symptoms, impact on work ability, possible workplace adjustments and job modifications should be considered. On the other hand we found that there is also a substantial group of individuals with PCS (77%) who were able to maintain work ability which may be related to their type of work or to the type of healthcare provided to cope or recover from PCS. Large scaled analysis focusing on healthcare utilization patterns in PCS combined with outcome measures such as work (dis)ability are needed to further assess the relationship between PCS and work.*

13) **Page 13: I am not sure what the authors mean by “To our knowledge there are no other studies using a data-driven approach to identify clinical subtypes of PCS.” The authors cite studies that identify clusters using data, and there are possible other studies e.g. (Bowyer, Huggins et al. 2023) that might be viewed as trying to find subtypes of PCS based on symptoms clustering. Please clarify.**

We appreciate this suggestion and have now elaborated on the suggested study in the Discussion as well (page 14, lines 16-18) and removed the phrase that to our knowledge there are no other studies using a EHR data and a data-driven approach to identify subtypes. When drafting the first version of the manuscript the suggested reference was not available yet and therefore it was not cited in the manuscript.

14) **Page 14: There should be more discussion of how representative the GP records really are of the population, perhaps supported by data. The data they have only covers 10% of the population and this may not be fully representative of all groups. Particularly those who are hard to reach. Biases arising from the use of administrative data are not fully appreciated see (Shaw, Harron et al. 2023)**

We have now described this in more detail in the manuscript (page 4, lines 26-28) and also referred to a report regarding the representativeness of the Nivel Primary Care database based on several aspects like: demographics of the patients, geographical location, type of general practice and urbanisation level. The added reference (which can also be found here: [Nivel Zorgregistraties Eerste Lijn: jaarcijfers 2022 en trendcijfers 2018-2022](https://www.nivel.nl/sites/default/files/bestanden/1004432.pdf)) is unfortunately in Dutch but the Tables and Figures regarding representativeness include descriptions in English.

15) **Finally, could you please include in or alongside supplementary table 1 (and possible other tables ) how the categories for age, education, income, and migration status were defined. I note that this is in the text of the main paper, but it would be helpful to have it in the same files as the table as well. Supplementary table 1 was introduced in the text before the variables were described.**

We have now added a description of the categories under Supplementary Table 1.

**Reviewer #2**

1) **In this study, the control group and PCS group were matched based on age, sex, and the COVID-19 period, but the exact method and criteria used for this matching are unclear. In addition, given that similar individuals were matched, how was the extent of matching assessed and validated. It might be helpful to refer to the following paper; PMID: 38802352 (Acute and post-acute respiratory complications of SARS-CoV-2 infection: population-based cohort study in South Korea and Japan).**

   The matching was done using R package ‘MatchIt’. As described in the manuscript (page 6, lines 12-16), each patient was matched to four controls based on age (nearest), sex and follow-up period. The characteristics of the matched control group are described in Supplementary Table 1. The matching was validated by comparing the control group to the patient group where no differences were found. We examined the paper suggested by the reviewer but in that paper a different matching method was used so we decided not to refer to that publication in the Manuscript.
2) **To enhance the clarity of Table 1, it may be beneficial to reorder the results listed. If the primary finding of this table is that there was a significant difference in frequency between the control and the narrow definition group, then it would be better to pair PCS and non-PCS side-by-side rather than in the current sequence.**

   We have now reordered the order of variables in Table 1 as suggested by the reviewer. Please find the revised Table 1 on page 19 of the Manuscript.
3) **Regarding Figure 1, are the top 10 most prevalent core symptoms defined under the "narrow" and "broad" criteria the same as those mentioned in the figure? Regarding Figure 1, are the top 10 most prevalent core symptoms defined under the "narrow" and "broad" criteria the same as those mentioned in the figure? Could you clarify the criteria used for sorting these symptoms? If the sorting is based on one of the definitions (e.g. narrow definition), it would be helpful to mention this in the method section or consider separating the graphs for the narrow and broad criteria.**

The top 10 of most prevalent symptoms, was based on the narrow definition unstratified by sex. As the top 10 based on the broad definition was fairly similar, we decided not to make separate graphs. This is now mentioned more explicitly in the manuscript (page 10, lines 14-15) and in the Figure legend.

4) **Figure 2 also requires the same clarification. Please refer to the comment 3.**

   For Figure 2 the sorting was based on the top 10 most prevalent symptoms in the 3 months survey are mentioned in the Corona Survey Cohort. We have now mentioned this in the Manuscript (page 10, line 24). and in the Figure legend.
5) **The GP-EHR data and the Corona survey cohort each employed different definitions for classification, yet the findings across these datasets do not align completely. For instance, concluding that "PCS mostly affects middle-aged females" based on the Corona survey cohort does not appear statistically significant. For that finding, was it only for GP-EHR data?**

There were more females with PCS in the Corona survey cohort (60%) but this was indeed not significantly different from the non-PCS group. We have now rephrased this stating that in the GP-EHR cohort we found that PCS mostly affects middle-aged females (page 15, lines 27-28).

6) **The statement in the Results section that "Most symptoms were reported at the same frequency or increased over time (3 to 6 months)" seems somewhat overstated. This observation appears to primarily apply to females.**

We agree that this was somewhat overstated and not clear. We have now rephrased this in the manuscript to be more clear (page 11, lines 2-4):

*“In females the reported frequency decreased for four symptoms while it increased for six symptoms. In males we found that the frequency decreased or stayed the same for seven symptoms while it increased for three symptoms.”*

7) **In my understanding, the study concluded on frequency alone, without any adjustment except for matching. It seems to lack a methodology to mitigate potential bias, and even then, an observational study requires a conservative approach to interpreting the findings, which I don't think was considered in this manuscript. It would be valuable to address these considerations more thoroughly in the paper.**

Observational studies like ours merely establish associations and cannot prove causality and biases can occur. We have therefore been careful when interpreting and presenting our results and avoided the suggestion of causality. In addition, in our approach we have taken several steps to mitigate possible biases especially when creating the classification in the EHR cohort. We have matched the COVID patients to four control patients, instead of one on one matching. We then compared the frequencies to determine which symptoms occurred more frequently in the COVID patients. We did this by comparing the top 10 most prevalent symptoms in COVID patients to the top 10 most prevalent symptoms in the matched control patients who were similar regarding age, sex and available follow-up data. By following this method we adjusted for these possible founders. In addition, we have adjusted the symptom list for symptoms for which the patients already consulted their GP before infection with COVID-19. By doing this we have adjusted for pre-existing health problems not related to COVID-19. This is all described in the Manuscript (page 6, lines 12-20).
