# Supplementary material for: Comparison of observational methods to identify and characterize post-COVID syndrome in the Netherlands using electronic health records and questionnaires
Source: PLoS One. 2025 Jan 29;20(1):e0318272. doi: 10.1371/journal.pone.0318272 (PMC11778627; doi:10.1371/journal.pone.0318272)
Supplement: Attachment — Submitted filename: PONE-D-24-29474_Response to Reviewers_part 2.docx [file pone.0318272.s006.docx]

Manuscript-ID: PONE-D-24-29474

Title: Comparison of observational methods to identify and characterize Post-COVID syndrome in the Netherlands using electronic health records and questionnaires

We would like to thank the reviewers for their thorough reading and constructive comments. This has helped us to further improve our manuscript. Below we addressed the comments and questions raised by the reviewers. We hope that the manuscript is now suitable for publication in PLOS ONE.

**Point-by-point response**

**Reviewer #1**

1. Thank you for including the two flowcharts in supplementary figure 1. The flowchart for the Corona Survey Cohort would be improved if it started with the 1,851 individuals who were invited to participate. That way the flowchart would cover a major potential source of bias, non-response.

   We have now added this to the flowchart of the Corona Survey Cohort in Supplementary Figure 1.
2. The inclusion of a discussion of why people with PCS stopped working on page 14 has also improved the paper. However, there is little discussion about the 0% of the non-PCS group stopping working. It is very unusual for nobody in a group to stop working, and I am wondering if this might be due to some form of bias or reverse causation.

   We agree that we did not discuss the 0% yet in the non-PCS group. We have now elaborated on this finding in the Discussion (page 14, lines 13-18) by adding the following text: “*In addition, further research which follows a patient group as well as a reference group, in this case the non-PCS group, over a longer period of time and also after the COVID pandemic is important as well. In this current study we only focused on a relatively short period and found very little changes regarding work in the non-PCS group (0% at 3 months and 1% at 6 months) which could be the result of an inclusion bias or related to the fact that ‘experiencing discomfort in daily living’ was part of the definition of PCS.”*
3. Thank you for clarifying that when you stated, “To our knowledge there are no other studies using a data-driven approach to identify clinical subtypes of PCS” in your original submitted manuscript that you are referring to studies based on Electronic Health Records. It was unclear form that draft. However, having clarified to what you are referring to the Bowyer et al 2023 reference may no longer be relevant.

Thank you for this clarification. After having read the manuscript by Bowyer et al. 2023 we do feel that it is still relevant to include this in the manuscript.

4. Could you please check ALL your references again. Some of them still do not seem to be complete. In particular, it is not clear what journal the Basharat et al (2022) and Case et al (2022) references are in. There may also be other mistakes.

   Some references where still incomplete because at the time of writing the first draft of the manuscript many publications were still in preprint. We have now carefully checked again all references.
5. Finally, while description of the data in the methods section is an improvement. That the data are “somewhat underrepresented like strongly urbanized regions” needs stating in the limitations section, with slightly more fluent English. More than 90 percent of the population of the Netherlands live in cites. The database may be representative of age and sex, but I would be very surprised if it was representative for ethnicity and some measures of socioeconomic disadvantage.

We have now rephrased the sentence regarding urbanisation level in the manuscript. More information regarding representativeness of Nivel-PCD can be found in the referred publication: Heins et al. 2023. Nivel-PCD is indeed not representative of ethnicity and socioeconomic status.

**Reviewer #2**:

1. In your response, it is mentioned that the control and patient groups were compared to verify the accuracy of the matching. Could you clarify where this is mentioned in the paper and where is the method used to test for no significant differences between the two groups?

   The validation of the matching was not yet mentioned in the manuscript. We have now mentioned this in the manuscript (page 6, lines 20-22).
2. In order to make a conclusion like this paper “Frequency rates differ between methods and data sources (15-33%) but characteristics of the affected individuals seem less associated as we found that PCS mostly affects middle-aged females”, two parts need to be clearly checked. First, a statistical test between PCS group and non-PCS group. Second, a test for the difference between the broad definition and the narrow definition. Only when the two parts are tested can we conclude that the frequency of PCS differed by definition (first step) and showed less associated characteristics for age and sex (second step). It is true that the mean values for age and frequency for sex seem to be similar, but this can only be concluded with a statistical test, and the results of the paper only showed a test between PSC and non-PCS. In observational studies, matching is a natural approach to address potential biases when examining associations. In this manuscript, however, the statement “we found that PCS mostly affects middle-aged females” seems to rely only on the EHR dataset, despite the Corona Survey Cohort results indicating no significant associations (age [p-value, 0.479], sex [p-value, 0.380]). Given these limitations, the author should at least present statistical test results to support the conclusion, or address these limitation in the manuscript.

   As presented in Table 1 we have conducted statistical testing between the PCS group and the non-PCS group for both the narrow and broad definition. In both comparisons we found that the PCS group is older compared to the non-PCS group and more often female. We however do agree that the differences between the PCS and non-PCS group were not significant in the Survey cohort. We therefore rephrased our conclusion in the manuscript (page 16, lines 3-5).
3. Please improve the resolution of the figures.

We have now improved the resolution of the figures.
