# Supplementary material for: Comparison of observational methods to identify and characterize post-COVID syndrome in the Netherlands using electronic health records and questionnaires
Source: PLoS One. 2025 Jan 29;20(1):e0318272. doi: 10.1371/journal.pone.0318272 (PMC11778627; doi:10.1371/journal.pone.0318272)
Supplement: Attachment — Submitted filename: PONE-D-24-29474_Response to Review_part 3.docx [file pone.0318272.s007.docx]

Manuscript-ID: PONE-D-24-29474

Title: Comparison of observational methods to identify and characterize Post-COVID syndrome in the Netherlands using electronic health records and questionnaires

Below we addressed the minor comments raised by the editor. We hope that the manuscript is now suitable for publication in PLOS ONE.

**Point-by-point response**

**1. Some of those infected with coronavirus suffer from post-COVID syndrome (PCS) -> Some of those infected with SARS-CoV-2 suffer**

We have now adjusted this throughout the manuscript.

**2. n=10.313 -> n=10,313 (comma)**

We have now adjusted this throughout the manuscript.

**3. coronavirus in 2021 => SARS-CoV-2 in 2021**

We have now adjusted this throughout the manuscript.

**4. Please add the definition of PCS in abstract method section.**

The aim of this study is to compare different definitions and classifications of PCS. It is therefore not possible to add *the* definition of PCS to the abstract because there is not one definition. As the Abstract may not exceed 300 words it is not possible to explain this all in the abstract. We did however try to clarify this better in the abstract by rephrasing the following sentence.

*In the GP-EHR data, we operationalized two classifications of PCS (based on symptoms and diagnosis recorded in GP-EHR data and healthcare utilization 3-12 months after acute infection) to calculate frequency and characteristics and compared this to the survey results.*

**5. Without a COVID-19 infection => without a SARS-CoV-2 infection. Please use the official term carefully. COVID infection (x) -> SARS-CoV-2 infection, Coronavirus (x) -> SARS-CoV-2, COVID pandemic (x) -> COVID-19 pandemic**

We have now adjusted this throughout the manuscript.

**6. In addition literature studies on symptoms that often co-occur do however also often show a cardiorespiratory cluster [42], a generalized-mood cluster [43], and a gastrointestinal cluster [18]. The current authors did not engage in any discussion regarding landmark studies on PCS. In fact, among the cited articles, there are no papers from top-tier journals.**
- <https://www.nature.com/articles/s41591-022-01689-3>
- <https://www.nature.com/articles/s41562-024-01895-8>
- <https://www.nature.com/articles/s41467-023-36223-7>
- <https://pubmed.ncbi.nlm.nih.gov/38802352/>

Thank you for these suggestions. Not all studies were available yet when drafting the first version of the manuscript. We have now added these important citations to the Discussion paragraph.
